# *KIF11* serves as a cell cycle mediator in childhood acute lymphoblastic leukemia

**DOI:** 10.1007/s00432-023-05240-w

**Published:** 2023-09-01

**Authors:** Liwen Zhu, Chuqin Chen, Meiyun Kang, Xiaopeng Ma, Xiaoyan Sun, Yao Xue, Yongjun Fang

**Affiliations:** 1 Department of Hematology and Oncology, Children’s Hospital of Nanjing Medical University, Nanjing, 220000 Jiangsu Province China; 2 Key Laboratory of Hematology, Nanjing Medical University, Nanjing, 220000 Jiangsu Province China

**Keywords:** Childhood acute lymphoblastic leukemia, WGCNA, *KIF11*, Cell cycle progression, Hub genes

## Abstract

**Objective:**

To identify key gene in childhood acute lymphoblastic leukemia (ALL) through weighted gene co-expression network analysis (WGCNA), and their enriched biological functions and signaling pathways.

**Methods:**

Array data of the GSE73578 dataset, involving 46 childhood ALL samples, were acquired from the Gene Expression Omnibus (GEO) database. Hub modules associated with childhood ALL were screened out by WGCNA. Enriched biological functions and signaling pathways were then identified by Gene Ontology (GO) and Kyoto Encyclopedia of Genes and Genomes (KEGG). Hub genes were selected by overlapping those between down-regulated genes in GSE73578, GSE4698 and the hub module. Guilt by association (GBA) was adopted to verify the function of the identified *KIF11* gene and to predict its target genes. Regulatory effects of *KIF11* on the proliferation and cell cycle progression of ALL in vitro were determined by cytological experiments.

**Results:**

WGCNA showed that the yellow module was the most relevant to childhood ALL treatment, containing 698 genes that were enriched in cell division, mitotic nuclear division, DNA replication and DNA repair, cell cycle, DNA replication and the P53 signaling pathway. The *KIF11* gene was screened out and predicted as a cell cycle mediator in childhood ALL. Knockdown of *KIF11* in ALL cells inhibited cell proliferation and arrested cell cycle progression in G_2_/M phase.

**Conclusions:**

The KIF11 gene is critical in the treatment process of childhood ALL, which is a promising therapeutic target for childhood ALL.

**Supplementary Information:**

The online version contains supplementary material available at 10.1007/s00432-023-05240-w.

## Introduction

As a prevalent malignant proliferative disease of the hematopoietic system, leukemia is caused by the blocked differentiation at a certain stage of hematopoietic stem cells (Pui et al. 2012; Malard and Mohty 2020). Acute lymphoblastic leukemia (ALL) is the most prevalent subtype of childhood leukemia, occupying 75% of leukemia cases and 43% of pediatric leukemia deaths globally. In China, there are over 10,000 newly diagnosed cases of ALL annually, the incidence of which has gradually on the rise (Yang et al. 2021). With great strides made on oncogene identification and immunotherapies, a valuable insight has emerged to guide the management of childhood ALL. Although the therapeutic efficacy and prognosis of childhood ALL has sharply advanced (Hunger and Mullighan 2015), the extremely high incidence and mortality of childhood ALL remain to be a huge challenge in the clinical practice (Wang et al. 2021; Duault et al. 2021).

Notably, tumorigenesis is a very complicated process involving various genes, pathways, and networks that closely interact with each other. Weighted gene co-expression network analysis (WGCNA) is a novel biological tool that transforms gene expression data into co-expression modules, which calculates the correlation coefficient value of gene expressions and thus causes a scale-free distribution of the network (Gu et al. 2019). It highlights signaling networks of interested clinical traits of a certain disease. Therefore, WGCNA provides more information about key genes and therapeutic targets through data mining (Liang et al. 2018), thus providing scientific references for developing individualized therapy and optimizing anti-cancer management.

In this study, WGCNA on the GSE73578 dataset involving 46 childhood ALL samples was performed. Hub modules associated with treatment of childhood ALL were identified, and the biological functions and pathways of genes enriched in hub module were analyzed. The *KIF11* gene was screened out by overlapping those between down-regulated genes in GSE73578, GSE4698, and the hub module, which was identified up-regulated in bone marrow samples of childhood ALL patients and corresponding cell lines. Moreover, the regulatory effect of *KIF11* on cell proliferation and cell cycle progression of ALL cells was explored. Our findings provide novel diagnostic and therapeutic targets for childhood ALL.

## Materials and methods

### Data source

Microarray expression profiling of GSE73578 and GSE4698 was obtained from the National Center for Biotechnology Information (NCBI) Gene Expression Omnibus (https://www.ncbi.nlm.nih.gov/geo/) database. All cohorts met the following criteria: (1) large sample size, (2) complete clinical information and microarray data, 3) fresh childhood ALL BM tissues for microarray analysis. The GSE73578 dataset consisted of gene expression profiling in bone marrow (BM) samples of 46 patients with childhood ALL before and after treatment using the GPL570 Affymetrix Human Genome U133 Plus 2.0 Array. The GSE4698 dataset consisted of 60 patients with the first relapse of childhood ALL using the GPL96 Affymetrix Human Genome U133A Array. The clinical information of the patients from GSE73578 dataset and GSE4698 dataset is shown in Supplementary Table 1. Annotating raw data, generating expression matrixes and matching probes targeting gene symbols were performed using the R package.

### Construction of WGCNA

The affy package (R version 3.4.3) was utilized to pre-process raw data of GEO database (.CEL file) (Langfelder and Horvath 2008), and they were normalized through the robust multi-array average (RMA). Outlier samples were identified and removed before co-expression analysis. The genes with an average normalized expression of 4 and above were selected and subjected to WGCNA. A hierarchical clustering tree (dendrogram) was depicted using the fashClust function. The soft-thresholding power was selected by the pickSoftThreshold function, which was a standard value in the scale-free topology network to ensure the power-law distribution of the established network. Through strengthening strong correlations and weakening weak correlations in a scale-free network feature, the soft-thresholding power contributed to eliminate errors as much as possible and made the results more characteristic of biological data. The scale-free topology fit index presented an exponential change. Therefore, a well correlation of *R*^*2*^ = 1 indicated a scale-free topological distribution of the data network.

### Identification of key module association with clinical features

The correlation between gene expression profile and module eigengene (ME) was defined as the module membership (MM), which was used to identify key modules (Ren et al. 2018). The log_10_ transformation of the *p* value (GS = lg*P*) was considered as gene significance (GS). Module significance (MS) was calculated by the mean GS of all genes in one module, and that with the highest MS was the most correlated one with clinical traits.

### Functional enrichment analysis of key module genes

GO and KEGG analyses were conducted to reveal biological functions and pathways enriched in genes of key module. Briefly, we analyzed functional annotation of selected genes in key modules using the online tool DAVID (Database for Annotation, Visualization and Integrated Discovery, https://david.ncifcrf.gov/). KEGG was analyzed using the online tool (https://www.genome.jp/kegg/) to illustrate the most enriched signaling pathways in the key module (Szklarczyk et al. 2017). The top 15 terms with a significant difference at *p* < 0.05 were visualized.

### Key gene identification and their validation

To confirm key genes in the hub module, differentially expressed genes (DEGs) between very early relapsed childhood ALL samples and late relapsed samples in the GSE4698 dataset, and those in BM samples before and after treatment in the GSE73578 dataset were analyzed using the limma R package with log_2_FC (fold change) >|2| and adj *p* value < 0.05. A Venn diagram was drawn by the online tool jvenn (http://jvenn.toulouse.inra.fr/app/example.html) to obtain the overlapping genes between downregulated genes and the hub module. The validation of key genes at the translational level was conducted using the Human Protein Atlas database (https://www.proteinatlas.org/).

### GBA

Putative functions of key genes were analyzed by GBA (Huarte et al. 2010). Briefly, Spearman’s rank correlation matrix of DEGs was created according to the normalized gene expressions of samples (counts per million). Initially, common genes associated with *KIF11* in the GSE73578 and GSE4698 dataset were subjected to GO and KEGG analysis. The Pearson relationship between *KIF11* and target genes was assessed by the Cor R package.

### Collection of clinical samples

Bone marrow samples were obtained from pediatric patients who were initially diagnosed as ALL and treated in the Children’s Hospital of Nanjing Medical University from January 2019 to December 2021. Exclusion criteria: (i) Patients with concurrent autoimmune disease, human immunodeficiency virus (HIV) or syphilis; (ii) History of immunosuppressive therapy within 1 month. Clinical characteristics of ALL were classified and diagnosed according to the guidelines of the Morphologic, Immunologic, and Cytogenetic and Molecular biologic Classification Technique. Clinical samples of controls were obtained from healthy volunteers or children who received bone marrow biopsy tests and were not diagnosed with hematological system diseases. A total of 19 ALL clinical samples (ranging 14–130 months of age, with 13 males and 6 females) were enrolled, while another cohort of healthy children as controls was labeled as control group (ranging 18–141 months of age, with 12 males and 7 females). The two groups were with gender- and age-matched. This study was performed in compliance with governmental policies and the Declaration of Helsinki, and approved by the Ethics Committee of Children’s Hospital of Nanjing Medical University (2016-SRFA-023). Written informed consent was obtained from guardians of all participants.

### Quantitative real-time PCR

Total RNAs of cells were extracted with TRIzol reagent (Invitrogen, Carlsbad, CA, USA), which were subjected to reverse transcription. RT-PCR was performed using the AceQ qPCR SYBR Green Master Mix Kit (Vazyme, China), with GAPDH as an endogenous control. The primer sequence of *KIF11* was: 5′-TCCCTTGGCTGGTATAATTCCA-3′ (forward) and 5′-GTTACGGGGATCATCAAACATCT-3′ (reverse).

### Cell culture and transfection

Jurkat (T line), Nalm-6 (B line), and 6T-CEM (T line) cell lines (Shanghai Cell Bank of Chinese Academy of Medical Sciences) were cultured in RPMI 1640 (Gibco, NY, USA) containing 10% fetal bovine serum (FBS) and 10% penicillin–streptomycin (Gibco, NY, USA). MAO cells (Kunming Cell Bank, Typical Culture Collection Committee, Chinese Academy of Sciences) were cultured in RPMI 1640 containing 20% FBS and 10% penicillin–streptomycin. All cells were routinely incubated in an incubator containing 5% CO_2_ at 37 °C.

*KIF11* shRNAs and negative control were synthesized by GeneChem, Shanghai, China. Sequences for shRNAs were as follows: sh*KIF11*-1, TACAGCAGAAATCTAAGGATA; sh*KIF11*-2, TTGAATAAGCCTGAAGTGAAT. Briefly, cells were transfected with plasmids for 72 h, and then incubated with 4 μg/mL puromycin for selection. Effectively transfected cells were assessed by fluorescence staining, followed by measurement of transfection efficacy via qRT-PCR and Western blot.

### Western blot

The total cellular protein was extracted using RIPA lysis buffer (Beyotime, China). Total proteins were subjected to sodium dodecyl sulfate–polyacrylamide gel electrophoresis (SDS-PAGE) and transferred onto an immobilon-P transfer membrane (Merck Millipore Ltd, Tullagreen, Carrigtwohill, Co. Cork IRELAND). After blockage of non-specific antigens, membranes cut into interested sizes and they were probed with primary antibodies at 4 °C overnight, and anti-rabbit or anti-mouse secondary antibodies on the other day. *KIF11* antibodies were purchased from Proteintech, USA. Band exposure was performed using the enhanced chemiluminescence reagents (Biosharp, Hefei, China) and normalized to gray values of *GAPDH*. After image exposure, the protein bands were analyzed using Image J software.

### CCK-8 (cell counting kit-8) assay

Cells were seeded in a 96-well plate with 8 × 10^3^ cells in 100 µl of culture medium per well. On day 0, 1, 2, 3, and 4, 10 µl of CCK-8 solution (APExBIO, Taiwan, China) mixed in 90 µl of fresh medium was added in each well at the fixed time point. The optical density (OD) was measured 4 h later using a microplate reader at 450 nm wavelength.

### EdU (5-ethynyl-2ʹ-deoxyuridine) assay

Cells were seeded in a 24-well plate with 3 × 10^5^ cells/well, which were incubated with EdU solution (1:3000) at 37 °C for 2 h. After 15-min fixation in 4% paraformaldehyde, 10-min permeabilization in 0.3% Triton X-100 at room temperature, and washing in 3% bovine serum albumin (BSA), cells were collected to stain the nuclei by 100 μl of Hochest33342 per well. EdU images were captured using an inverted fluorescence microscope (OLYMPUS-IX73).

### Flow cytometry

Cells were washed in PBS twice, incubated with RNase A and stained with propidium iodide (MULTI SCIENCES, China) in the dark. Cell cycle progression was determined by flow cytometry using FlowJo software.

### Statistical analysis

Statistical analysis was performed by Graphpad prism 9.0 and R language (version 3.6). Data were presented as the mean ± standard deviation ($\overline{x }$±SD) of at least three independent experiments. Differences between groups were compared by the Student’s* t* test. Two-sided *p* < 0.05 was considered as statistically significant.

## Results

### Construction of WGCNA and identification of hub module

**Fig. 1:**
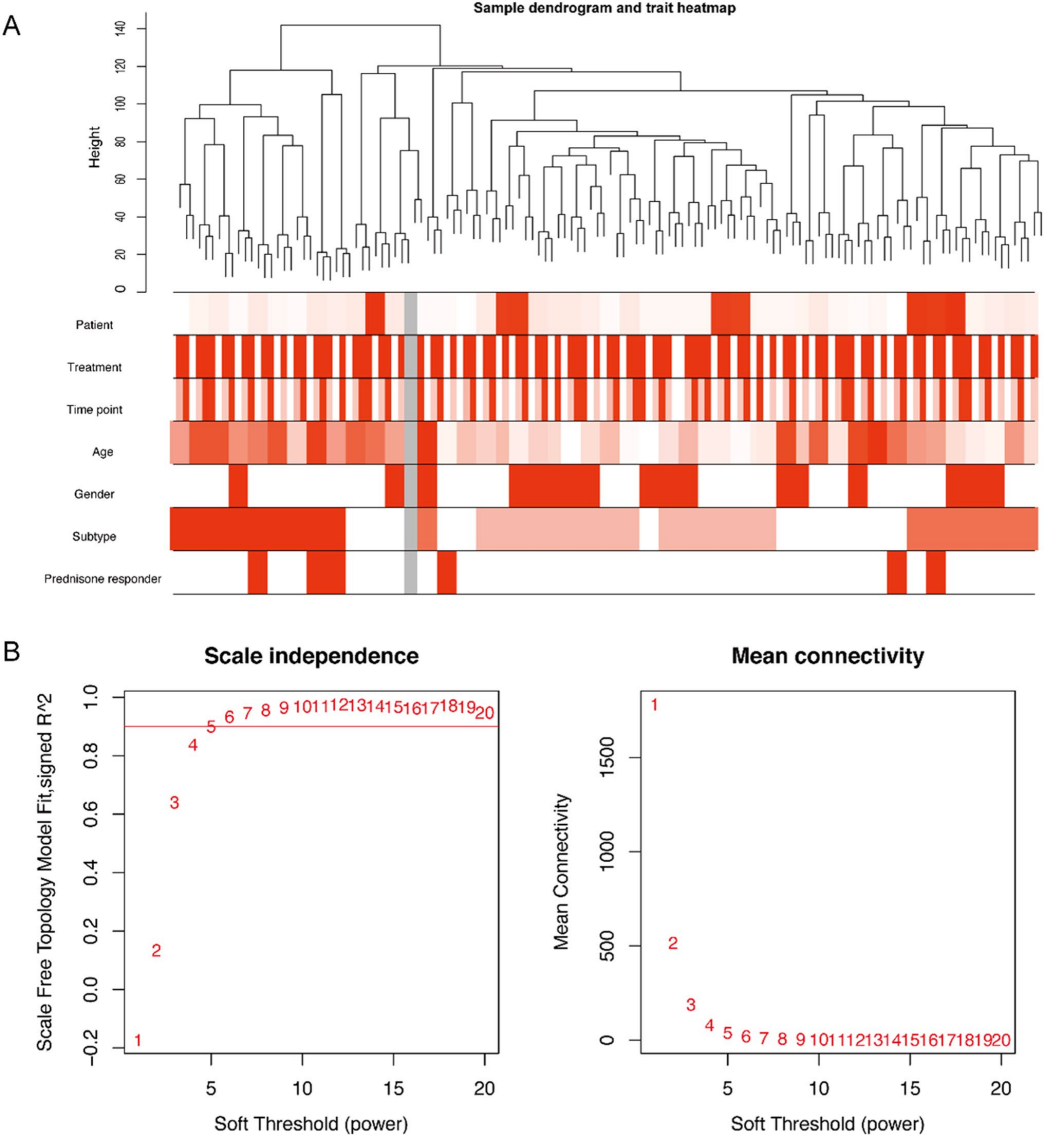
Clustering dendrogram and determination of soft-thresholding power in the WGCNA. **A** A cluster of 46 ALL patients from the GSE73578 dataset. The top 5000 genes with the highest standard deviation values were used for WGCNA. The color intensity was proportional to treatment, time point, sex, age, subtype, and prednisone responder. **B** Analysis of the scale-free fit index for various soft-thresholding powers (*β*) and the mean connectivity for various soft-thresholding powers

**Fig. 2:**
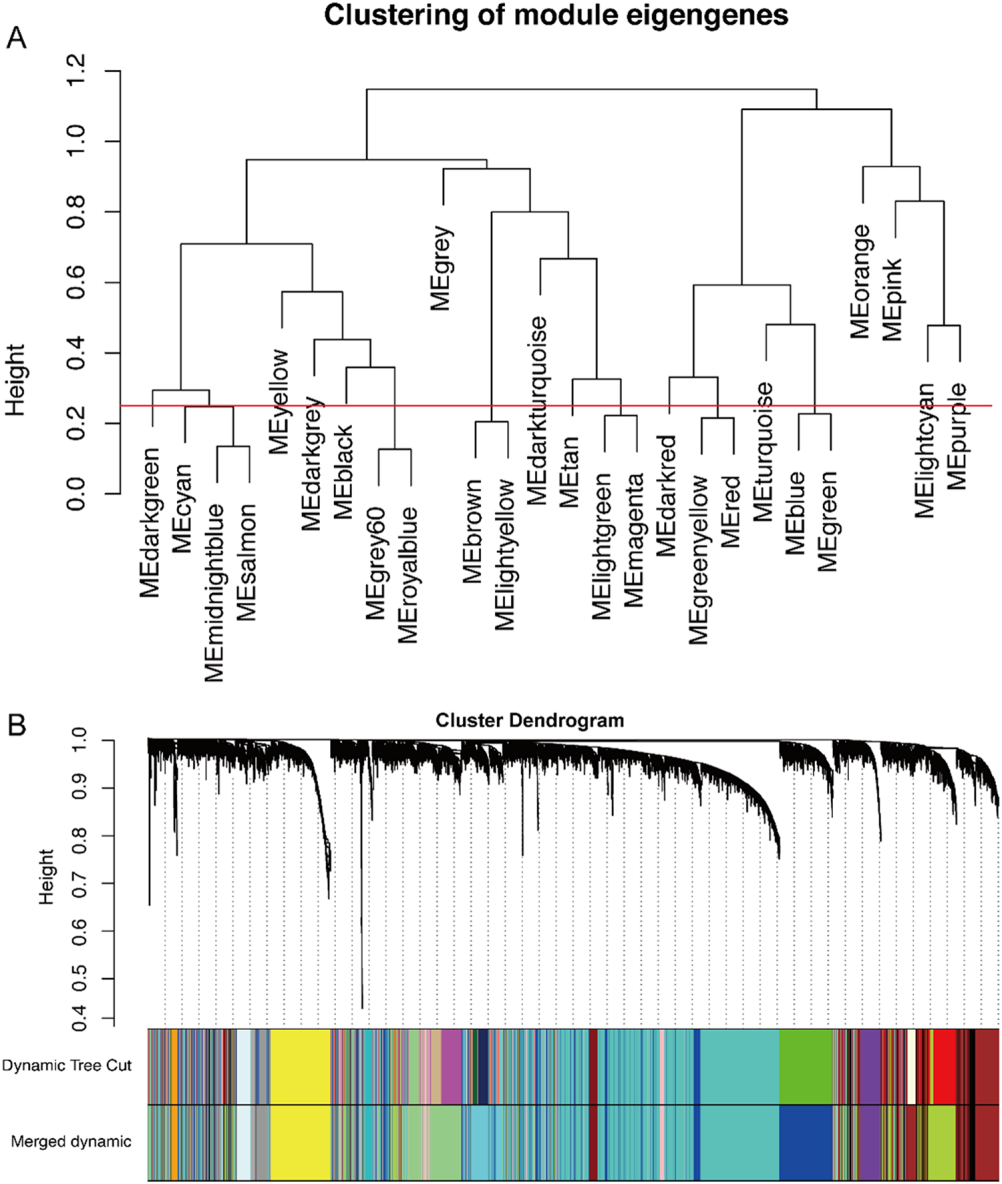
Construction of co-expression modules by WGCNA package in R. **A** A hierarchical clustering of module eigengenes that summarized the modules found in the clustering analysis. Branches of the dendrogram (the meta-modules) group together eigengenes that were positively correlated. **B** The cluster dendrogram of genes in GSE73578. Each branch in the figure represented one gene, and every color below represents one co-expression module. Gray module color was a reserved one for genes that were not part of any module. A total of 16 merged co-expression modules were obtained by merging similar modules when the MEDissThres was set as 0.25

There were 20,462 genes in the GSE73578 database, and those with an average normalized expression of 4 and above were subjected to the construction of a co-expression network using the R package. It is shown that the gene expression data of 46 childhood ALL samples were assigned into 2 clusters of the dendrogram. The trait heatmap showed that the clinical data of all patients were completely documented, and these traits could be used for WGCNA (Fig. 1A). The network topology of β ranging from 1 to 20 was analyzed. To create a hierarchical clustering tree, the power value of 5 was set as the lowest limit for the scale-free topology to ensure the scale independence > 0.85 and mean connectivity close to 0 (Fig. 1B). Co-expression modules at MedissThres of 0.25 were merged, and the eigengene adjacency heatmap demonstrated the relationship between modules (Fig. 2A). At last, 16 modules were recognized by the dynamic tree cut and labeled with different colors (Fig. 2B). Genes that were unable to be included in any modules were put into the gray module, which were removed in the subsequent analysis.

### Module-clinical trait correlation

**Fig. 3:**
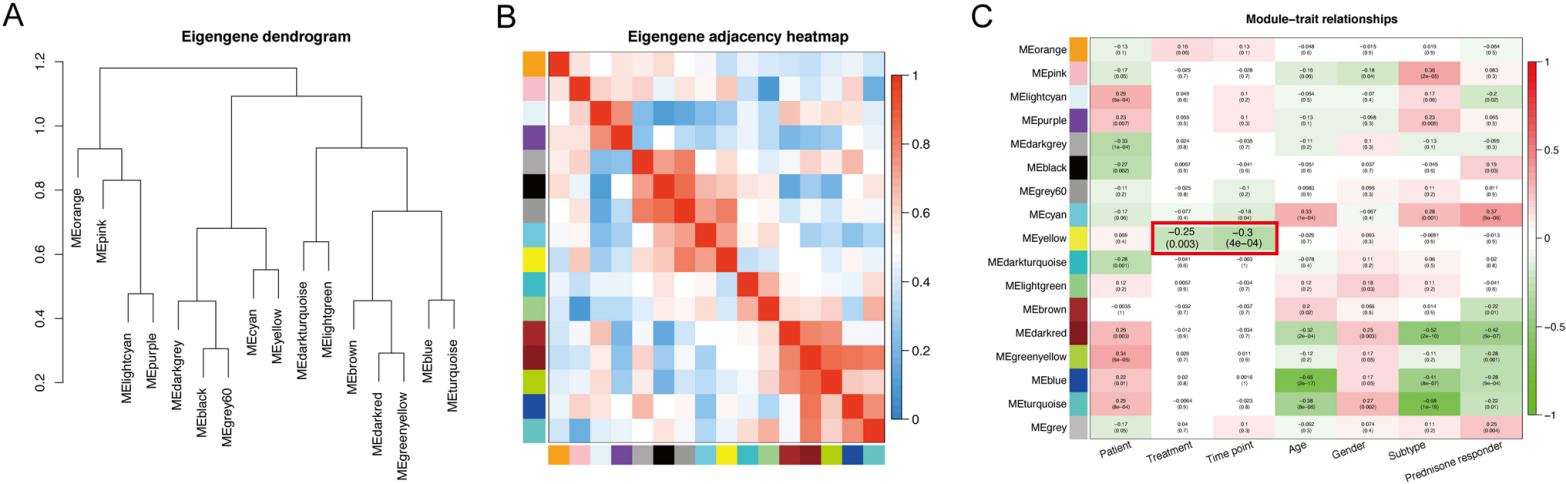
Gene modules identified by WGCNA. **A** Dendrogram of consensus module eigengenes obtained by WGCNA on the consensus correlation. **B** Heatmap plot of the adjacencies of modules. Red represented positive correlation and blue represented negative correlation. **C** Relationships of consensus module eignegenes and clinical traits. Each row corresponded to a module eigengene. The module name was shown on the left side of each cell, each column corresponded to a clinical feature. Each cell line contained the corresponding correlation in the first line and the *P* value in the second line. The table was color-coded by correlation according to the color legend. Intensity and direction of correlations were indicated on the right side of the heatmap, in which red and green represented a positive and negative correlation, respectively

The co-expression similarity of modules was determined by calculated and clustered MEs (Fig. 3A, B). It is shown that the gene in the yellow module (MEyellow) was the most negatively relative module to the treatment (*R = *− 0.25, *P* = 0.003) and time point (*R = *− 0.3, *P* = 4e^−4^) compared with others (Fig. 3C). Accordingly, the yellow module was set as the real clinically significant module, which was analyzed further.

### Biological functions of genes in the hub module

**Fig. 4:**
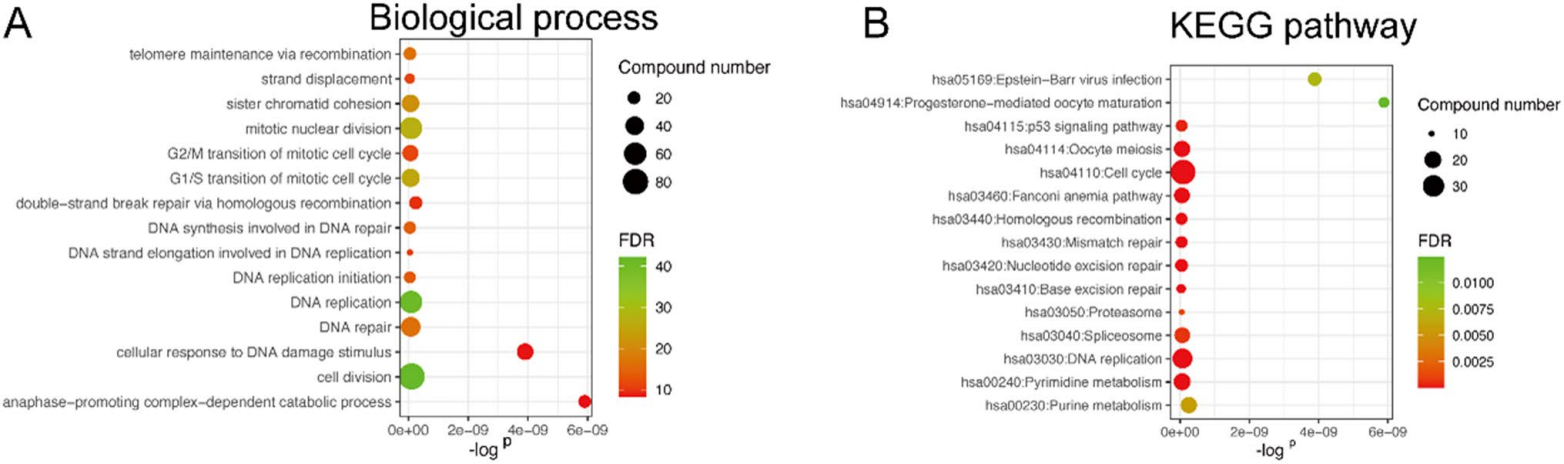
Functional enrichment analysis for genes in the object module. **A** GO enrichment analysis of genes in the yellow module. **B** KEGG enrichment analysis of genes in the yellow module

To illustrate biological functions of 698 genes in the yellow module, we performed GO and KEGG pathway enrichment analyses. GO enrichment of BP was conducted by DAVID, and the detailed information was given in Supplementary Table 3. The results showed that the genes in this module were mainly enriched in the biological processes of cell division, mitotic nuclear division, DNA replication, and DNA repair (Fig. 4A). Based on KEGG database, the genes were predominantly enriched in cell cycle progression, DNA replication, and *P53* signaling pathway (Fig. 4B, Supplementary Table 4).

### Key gene identification

**Fig. 5:**
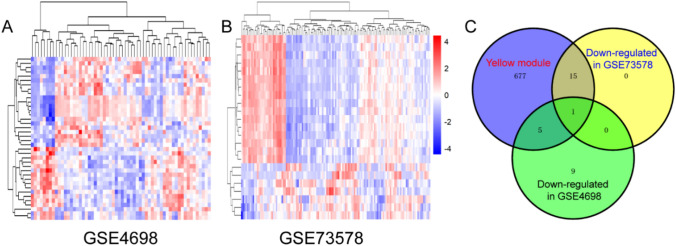
Key gene identification. **A** Heatmap of different genes between very early relapsed ALL compared with late relapsed disease in GSE4698. **B** Heatmap of different genes between pre- and post-treatment in GSE73578. Blue and red represented downregulation and upregulation, respectively. **C** Identification of key gene between the downregulated genes in GSE73578, in GSE4698 and the yellow module by overlapping them

To identify key genes in the yellow module, DEGs between very early relapsed childhood ALL samples and late relapsed samples in the GSE4698 dataset and those in BM samples before and after treatment in the GSE73578 dataset were screened out and depicted in the heatmap (Fig. 5A, B). A Venn diagram was drawn by overlapping downregulated genes among GSE4698, GES73578, and the yellow module. Finally, the *KIF11* gene was screened out (Fig. 5C).

### *KIF11* is a cell cycle mediator in childhood ALL

**Fig. 6:**
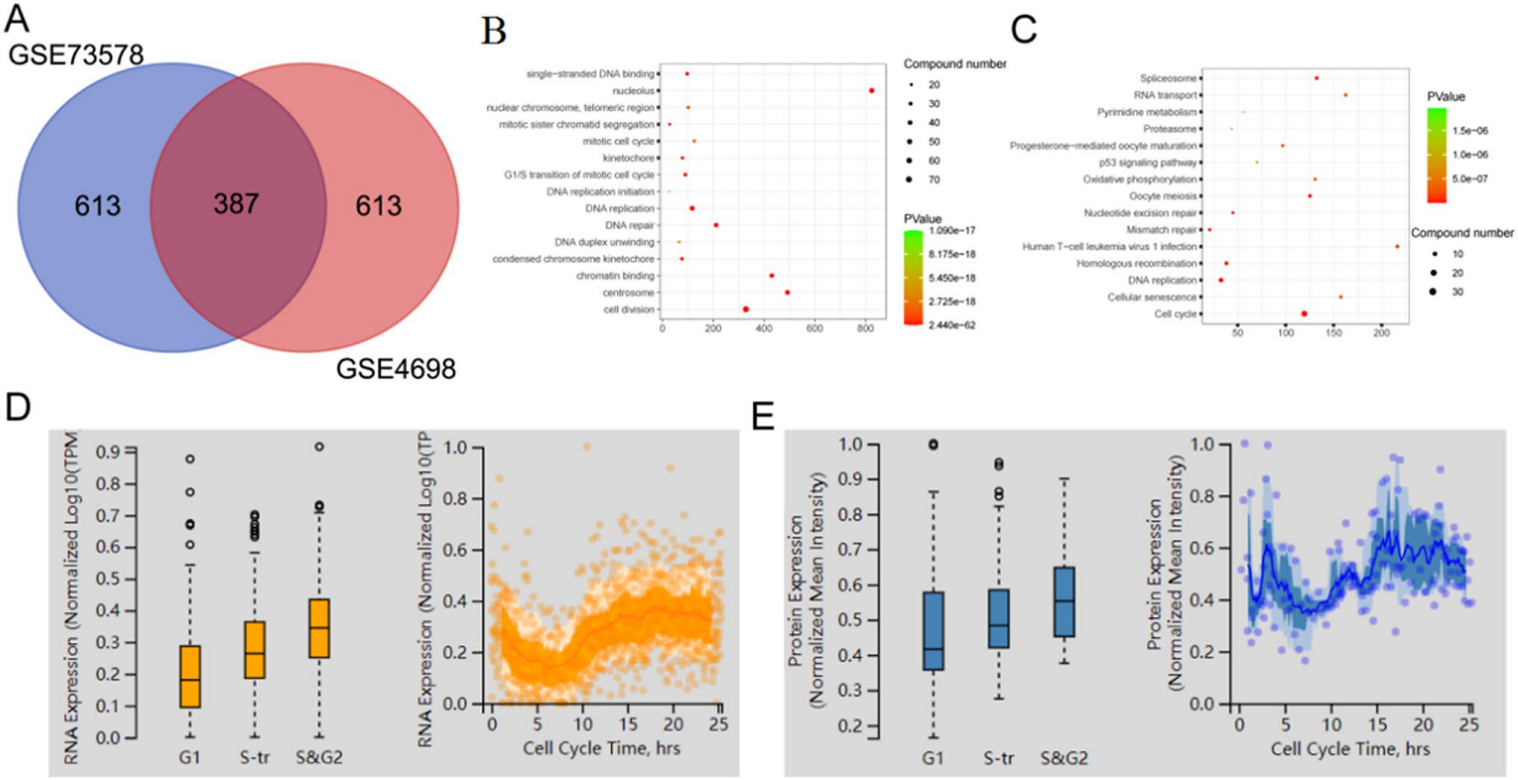
Inferring the functions of KIF11 by integrative bioinformatics analyses. **A** Venn diagram of the top 1000 positive correlated genes with KIF11 in GSE73578 and GSE4698. **B** GO analysis of genes associated with *KIF11* in GSE73578 and GSE4698. **C** KEGG analysis of genes associated with *KIF11* in GSE73578 and GSE4698. **D** The mRNA expression of *KIF11* in different cell cycle phases. **E** The protein expression level of *KIF11* in different cell cycle phases

We subsequently analyzed *KIF11* function based on known functions of genes that are co-expressed with it by GBA. The Pearson correlations between *KIF11* and associated genes were computed. 387/1,613 (24%) of *KIF11*-associated genes were shared in the GSE4698 and GSE73578 dataset (Fig. 6A). Next, we performed GO and KEGG analysis on the common KIF11-associated genes in the GSE4698 and GSE73578 dataset. Enrichment analyses revealed that cell division and cell cycle pathway were mainly enriched in *KIF11*-associated genes (Fig. 6B, C). Moreover, translational expressions obtained from the Human Protein Atlas database also demonstrated the expression status of *KIF11*. The mRNA and protein expressions of *KIF11* in different cell cycle phases were measured, and they were upregulated in phase S and G_2_ (Fig. 6D, E). It is suggested that *KIF11* served as a cell cycle mediator in childhood ALL.

### Possible mechanism for *KIF11* in regulating the development of childhood ALL

**Fig. 7:**
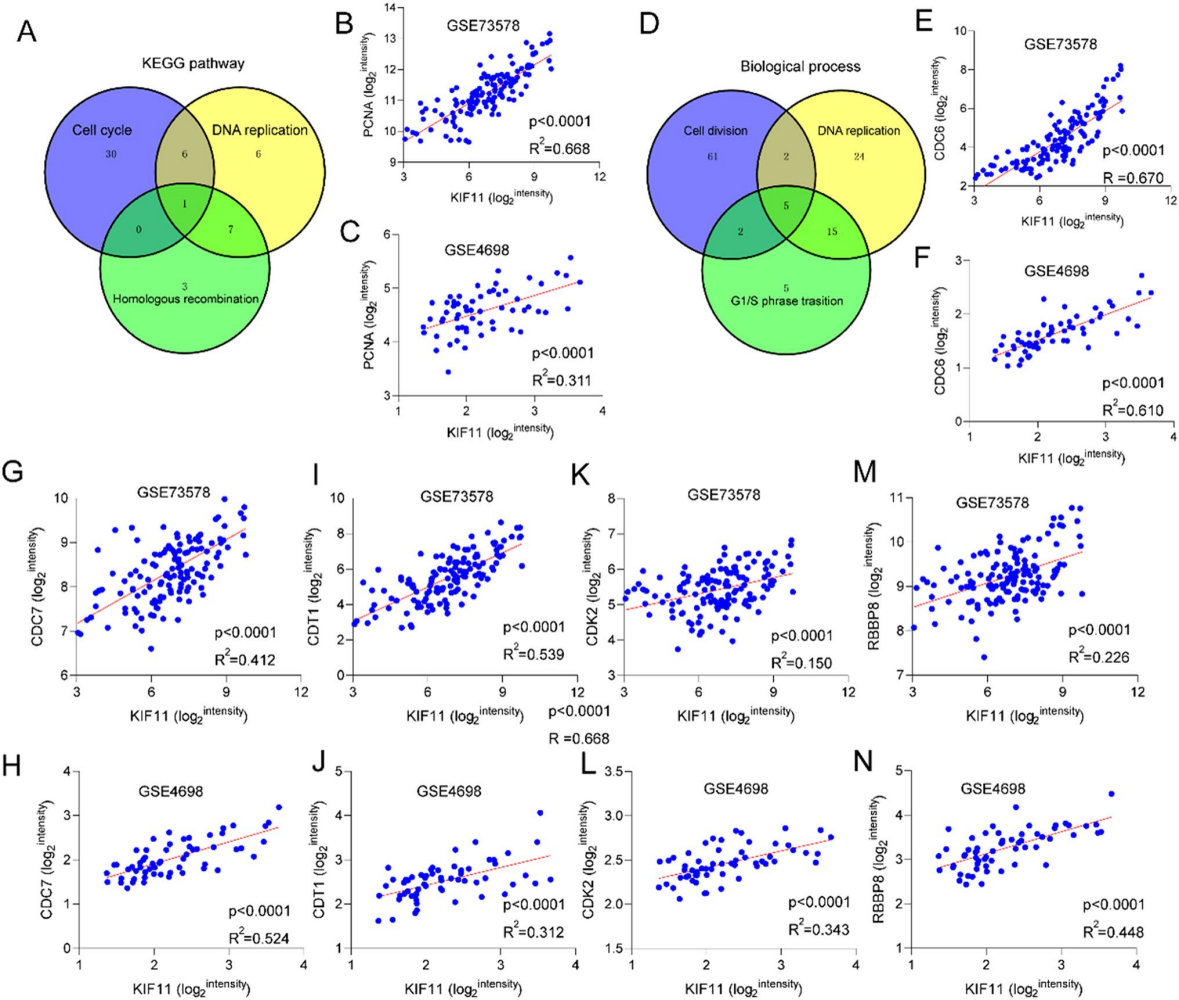
Correlation between the expression of *KIF11* and key factors in ALL. **A** Venn diagrams of genes in cell cycle, DNA replication and homologue recombination pathways. **B**, **C** *KIF11* expression is positively correlated with *PCNA* in GSE73578 and in GSE4698 respectively. **D** Venn diagrams of genes in cell division, DNA replication and G_1_/S phase transition biological progress. *KIF11* expression is positively correlated with *CDC6* (**E**, **F**), *CDC7* (**G**, **H**), *CTD1* (**I**, **J**), *CDK2* (**K**, **L**), *RBBP8* (**M**, **N**) in GSE73578 and in GSE4698, respectively

To narrow the target genes that regulated by *KIF11* in regulating cell cycle pathways among the 387 genes, the Venn diagram method was enrolled in our analysis. Proliferating cell nuclear antigen (*PCNA*) was the overlapped gene among cell cycle, DNA replication, and homologous recombination KEGG pathway (Fig. 7A). The Pearson analysis revealed a strong positive correlation between expression levels of *PCNA* and *KIF11* in the GSE73578 and GSE4698 datasets (Fig. 7B and C). The same method was enrolled to obtain the overlapped genes among cell division, DNA replication, and G_1_/S phase transition, and 5 common genes in three biological processes were identified (Fig. 7D). Cell division cycle 6 (*CDC6*), Cell division cycle 7 (*CDC7*), C-terminal domain 1 (*CDT1*), cyclin-dependent kinase 2 (*CDK2*), and RB-binding protein 8 (*RBBP8*) were positively associated with *KIF11* in both the GES73578 and GSE4698 datasets (Fig. 7E–N). It is suggested that *KIF11* may influence the treatment of childhood ALL via regulating expression levels of *PCNA*, *CDC6*, *CDC7*, *CDT1*, *CDK2*, and *RBBP8*.

### Knockdown of *KIF11* inhibits in vitro cell proliferation and arrests cell cycle progression in G_2_/M phase of ALL cells

**Fig. 8:**
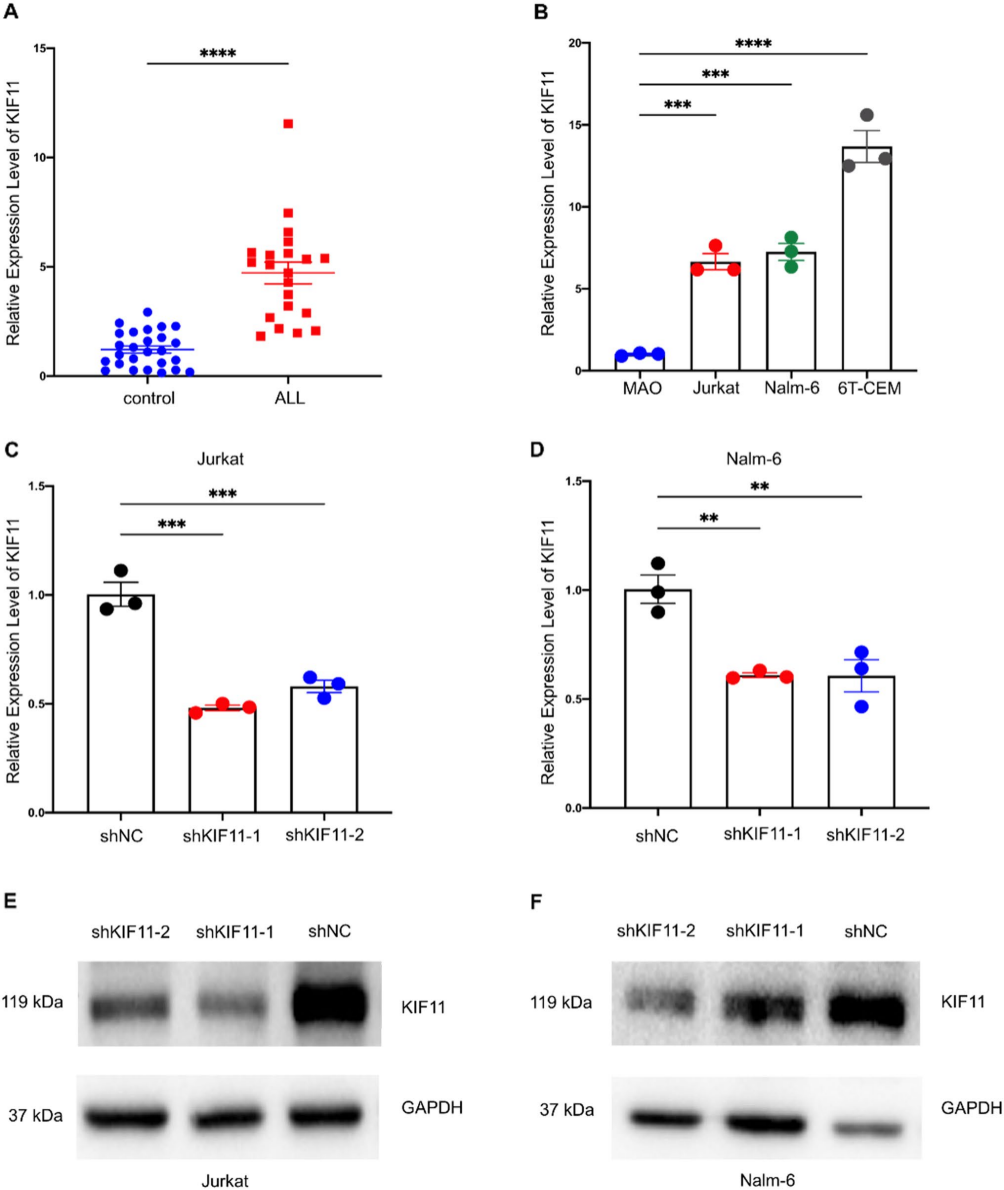
Verification of *KIF11* expression. **A** Relative levels of *KIF11* in bone marrow samples of ALL children and negative controls (*n = *24). **B** Relative levels of *KIF11* in ALL cell lines. **C**, **D** Transfection efficacy of sh*KIF11*-1 and sh*KIF11*-2 in Jurkat and Nalm-6 cells detected by qRT-PCR. **E**, **F** Transfection efficacy of sh*KIF11*-1 and sh*KIF11*-2 in Jurkat and Nalm-6 cells detected by Western blot. **P < 0.01, ***P < 0.001, ****P < 0.0001

**Fig. 9:**
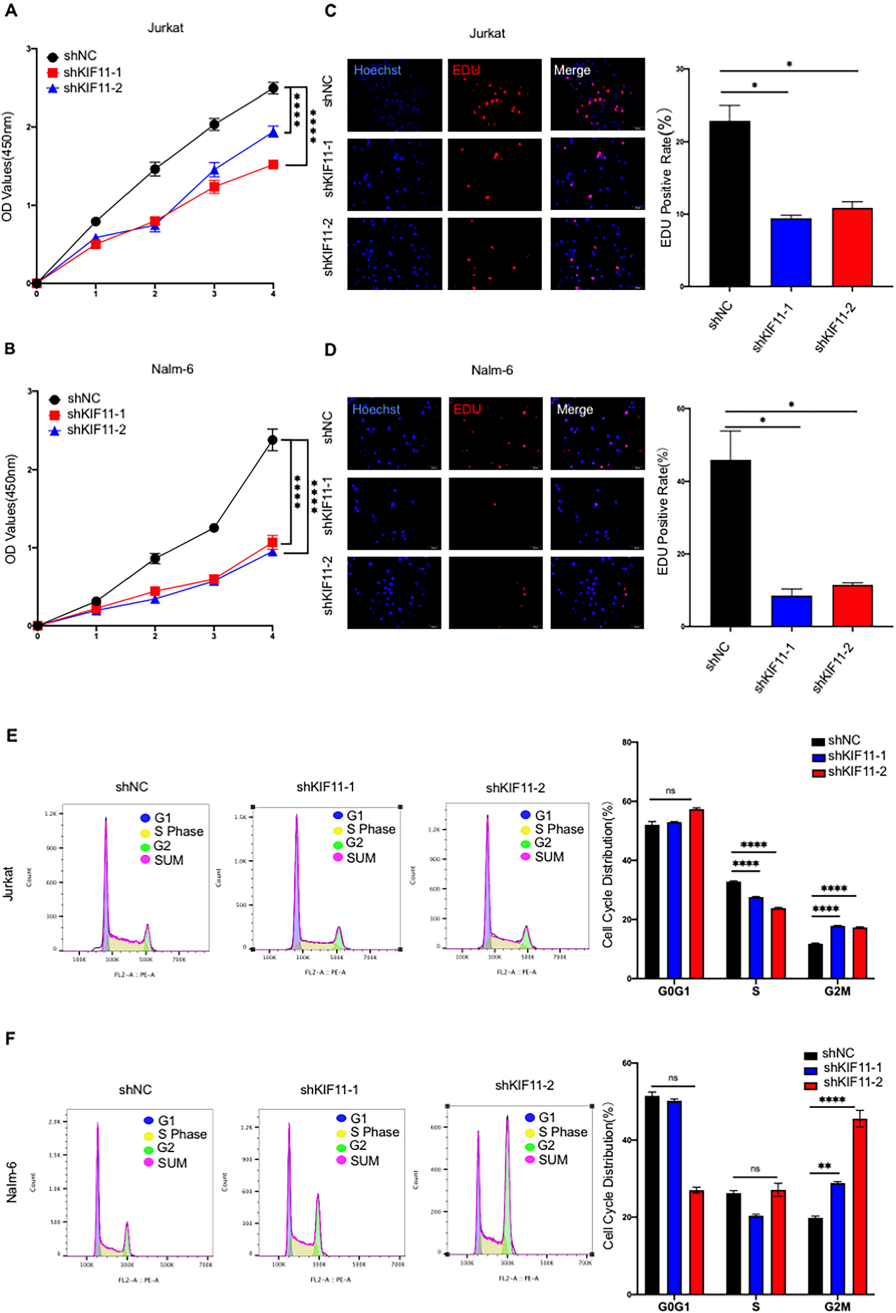
Knockdown of *KIF11* inhibits in vitro cell proliferation and arrests cell cycle progression in G_2_/M phase of ALL cells. **A**, **B** Cell viability in Jurkat and Nalm-6 cells detected by CCK-8 assay. **C**, **D** EdU-positive ratio in Jurkat and Nalm-6 cells. **E**, **F** Cell cycle progression of Jurkat and Nalm-6 cells detected by flow cytometry. Ns, no significant difference, *P < 0.05, **P < 0.01, ****P < 0.0001

To measure the expression of *KIF11* in ALL samples, real-time PCR was performed to detect the mRNA expression of *KIF11* in BM samples of 19 ALL children and 19 healthy volunteers. *KIF11* mRNA in ALL patients was significantly higher than that in the control group (*P* < 0.05, Fig. 8A). Correlation analysis revealed that *KIF11* expression is consistent across different ages, genders, and other prognostic factors in ALL. (Supplementary Table 4). Compared with that of normal lymphocytes, *KIF11* was highly expressed in three ALL cell lines (Fig. 8B). Then, we verified the transfection efficiency of two *KIF11* shRNAs (sh*KIF11*-1 and sh*KIF11*-2) in Jurkat and Nalm-6 cells by qRT-PCR, both of them were qualified (Fig. 9C, D). The results of real-time PCR and Western blotting indicated that the expression level of *KIF11* was significantly downregulated by transfection of shKIF11-1 or shKIF11-2 (Fig. 8E, F). Later, the regulatory effect of *KIF11* on the proliferation of ALL cells was assessed by CCK-8 and EdU assay. Compared with those transfected with shNC, Jurkat and Nalm-6 cells with *KIF11* knockdown presented a significantly lower cell viability (*P* < 0.01, Fig. 9A, B) and EdU-positive rate (*P* < 0.05, Fig. 9C, D), suggesting that knockdown of *KIF11* inhibited the proliferation of ALL cells. Moreover, the flow cytometry analysis illustrated that knockdown of *KIF11* remarkably arrested ALL cells in G_2_/M phase (*P* < 0.01, Fig. 9E, F). It is concluded that *KIF11* was capable of mediating cell proliferation and cell cycle progression of ALL.

## Discussion 

Superb than conventional bioinformatic methods analyzing DEGs in disease samples, WGCNA creates a network involving gene modules to analyze the correlation between their enriched functions and clinical traits. WGCNA, serving as a newly emerged tool, has been widely applied to bioinformatic analyses. Sun Z et al. identified four key oncogenes in prostate cancer (PCa) via WGCNA (Sun et al. 2021). MAGI2 identified by WGCNA is found to mediate cytoskeletal rearrangement of podocytes, the loss of which causes proteinuria (Zhou et al. 2019) and chronic kidney disease (Zuo et al. 2018). We believed that WGCNA is of great significance in searching for key biomarkers in human diseases, especially cancers.

ALL is the leading cause of pediatric cancer (Malard and Mohty 2020; Kadan-Lottick et al. 2003), the cure rate of which has been growingly risen with the advanced made on contemporary combination chemotherapy and refined risk stratification (Hunger et al. 2012). Nevertheless, some ALL children are unable to benefit from the treatment. It is necessary to develop biomarkers for effectively managing childhood ALL.

In the present study, multiple bioinformatic methods including WGCNA, functional enrichment analyses, and GBA analysis were utilized. The GSE73578 dataset was subjected to WGCNA and classified into 16 modules. A negative correlation was yielded between the yellow module and the treatment of ALL, and genes in which were mainly enriched in cell division and cell cycle by GO and KEGG analyses. The cell cycle progression contributes to influence cell division, differentiation, and death (Liu et al. 2022; Matthews et al. 2022). In the present study, *KIF11* was identified as the key target for influencing childhood ALL, which, analyzed by GBA, was confirmed as a cell cycle mediator. To further predict the possible mechanisms of *KIF11* in ALL, the correlation between *KIF11* and its protein-encoding genes in ALL samples was assessed. Some previously identified key factors in the development of ALL were found strongly correlated with *KIF11*, including the cell cycle regulating factor *PCNA*, *CDC6*, *CDC7*, *CDT1*, *CDK2*, and *RBBP*8. *KIF11* was upregulated in BM samples of childhood ALL patients and corresponding cell lines. In vitro experiments further confirmed that knockdown of *KIF11* in ALL cells inhibited cell proliferation and arrested cell cycle progression in G_2_/M phase.

*KIF11* belongs to the kinesin superfamily that is functional in mediating positioning and separation of chromosome, bipolar spindle assembly, and facilitating mitosis (Hata et al. 2019; Owens 2013; Blangy et al. 1995). *KIF11* is usually upregulated in malignant tumors (Zhou et al. 2019). It is reported that *KIF11* is capable of predicting poor prognosis of hepatocellular carcinoma, the expression level of which is correlated with tumor staging (Shao et al. 2021). In vitro evidences have validated the role of *KIF11* in mediating migratory capacity and angiogenesis of tumor cells. Intracellular localization of *KIF11* in interphase cells is suggested as a biomarker for predicting the prognosis of hormone-naive PCa. Nuclear level of *KIF11* indicates a poor overall survival and high risk of aggravation in metastatic castration-resistant PCa. Involved in the G_2_/M phase transition, *KIF11* contributes to influence tumor progression by regulating cell cycle checkpoints.

Its prognostic value and functions, however, have not been clearly elaborated. Our study found six genes (*PCNA, CDC6, CDC7, CDT1, CDK2*, and *RBBP8*) that were most significantly linked with expression levels of key genes and tumor progression. For example, *PCNA* is a ring-shaped homo-trimeric DNA clamp that acts on DNA replication and DNA repair (Strzalka and Ziemienowicz 2011; Peng et al. 2019). It is upregulated in lung cancer and stimulates malignant phenotypes of lung cancer cells by upregulating *STAT3* (Wang et al. 2018). *CDC6* and *CDC7,* members of the cell division control protein family, are of significance in cell division, cell cycle checkpoint and recombination signaling pathways. Recent studies have unveiled their proto-oncogenic activity and promising novel biomarker and druggable target in cancers (Liu and Huang 2022; Lim and Townsend 2020; Montagnoli et al. 2010). *CDT1* initiates DNA replication, which contributes to genome stability via mediating cell cycle progression and DNA damage response. Wang et al. (2022) reported the role of *CDT1* in triggering PCa cell metastasis by driving cell cycle and EMT via the PI3K/AKT/GSK3β axis. Upregulated *CDT1* in childhood ALL promotes cell proliferation, invasion, and migration through activating EMT (Ding et al. 2021). *CDK2* is widely involved in cell cycle progression, which, alongside its regulatory subunits are dysregulated, showing tumor-promoting features (Shi et al. 2021). *CDK2*-mediated TNFα upregulation induces in vitro apoptosis of *TP53*-null acute myeloid leukemia (AML) and BCR/ABL-positive ALL (Tadesse et al. 2020). *RBBP8* is reported to be involved in G_2_/M cycle checkpoints in DNA double-stranded breaks (Mozaffari et al. 2021). Through inducing the histone deacetylation of *p21* promoter and inhibiting *p21* transcription via *CtBP* and *BRCA1*, *RBBP8* serves a vital regulator in gastric cancer (Yu et al. 2020).

*KIF11* has been well concerned as a promising target for mitosis. Many phase I–II clinical trials have investigated *KIF11*-targeting agents (Holen et al. 2011; Hansson et al. 2020; Infante et al. 2017). For example, filanesib (Arry-520), a *KIF11* inhibitor, is validated as a promising agent in phase II clinical trials of multiple myeloma, which is effective in both monotherapy and combination therapy with proteasome inhibitors (Hansson et al. 2020; Hernández-García et al. 2017; Algarín et al. 2020). Therapeutic efficacy of *KIF11* inhibitors has been previously reported. SB-743921 is also a specific *KIF11* inhibitor. Treatment of SB-743921 in clear cell renal cell carcinoma cells remarkably inhibits proliferative and migratory capacity, as well as the EMT process, and stimulates cell apoptosis (Jin et al. 2019). Ispinesib (SB-715992) is a highly specific *KIF11* inhibitor, which widely exerts the anti-tumor activity through maintaining the complete response (Myers and Collins 2016). Two complete and two partial responses are achieved in 6 childhood ALL xenografts intervened by ispinesib (Mills et al. 2017). Consistently, our results also indicated the promising application of *KIF11* in the treatment of childhood ALL.

Through literature review, only one study reported the involvement of *KIF11* in the development of B-cell leukemia (Hansen and Justice 1999). Its prognostic value and functions, however, have not been clearly elaborated. We for the first time demonstrated the role of *KIF11* as a cell cycle mediator in ALL determined by WGCNA, which was further confirmed in in vitro experiments. Some limitations should be noted. First, clinical data, especially the survival data were scant in the GSE73578 and GSE4698 datasets, which may be attributed to the high 5-year event-free survival (Yang et al. 2021). We are now collecting follow-up data of ALL patients for the following in-depth analysis. Second, we only verified the in vitro effects of *KIF11* on ALL cells, the in vivo functions, however, were not confirmed in animal models. Thirdly, the exact mechanism underlying the regulatory effect of *KIF11* on the proliferation and cell cycle progression of ALL remained unclear, which will be explored in future. In summary, combined with the result of bioinformatic analyses and in vitro experiments, our study found that *KIF11* exerted a key role in the progression of childhood ALL, suggesting that *KIF11* was a potential therapeutic target. Therefore, this study offers new ideas for the disease prevention, diagnosis and treatment of childhood ALL.

## Conclusions

Taken together, *KIF11* is screened out for childhood ALL through bioinformatic methods and in vitro experiments, which may serve as a viable molecular biomarker or therapeutic target in childhood ALL. The mechanism underlying the regulatory effect of *KIF11* on childhood ALL needs to be explored in future.

## Supplementary Information

Below is the link to the electronic supplementary material.

Supplementary file1 (DOCX 19 KB)

Supplementary file2 (DOCX 33 KB)

Supplementary file3 (DOCX 32 KB)

Supplementary file4 (DOCX 20 KB)

## Data Availability

The datasets used and/or analyzed in the current study are available from the corresponding author upon reasonable request.
