# Supplementary material for: KIF11 serves as a cell cycle mediator in childhood acute lymphoblastic leukemia
Source: J Cancer Res Clin Oncol. 2023 Sep 1;149(17):15609–22. doi: 10.1007/s00432-023-05240-w (PMC10620298; doi:10.1007/s00432-023-05240-w)
Supplement: Supplementary file 1 — Supplementary file1 (DOCX 19 KB) [file 432_2023_5240_MOESM1_ESM.docx]

**Supplementary Table 1. Baseline characteristics of childhood ALL patients in the datasets.**

| Baseline characteristics | GSE73578  (n=46) | GSE4698  (n=60) | Sample  (n=19) |
| --- | --- | --- | --- |
| Gender (n, %) |  |  |  |
| Male | 28 (60.9) | 41 (68.3) | 13 (68.4) |
| Female | 18 (39.1) | 19 (31.7) | 6 (31.6) |
| Age (years) |  |  |  |
| <1 or ≥10, n (%)  ) | 12 (26.1) | 28 (46.7) | 3 (15.8) |
| ≥1 and <10, n (%) | 34 (74.0) | 32 (53.3) | 16 (84.2) |
| Risk stratification (n, %) |  |  |  |
| SR | 10 (21.7) | 9 (15.0) | 9 (47.4) |
| IR | 27 (58.7) | 22 (36.7) | 10 (52.6) |
| HR | 8 (17.4) | 10 (16.7) | 0 (0.0) |
| Immunophenotype (n, %) |  |  |  |
| T-ALL | 9 (19.6) | 8 (13.3) | 3 (15.8) |
| B-ALL | 37 (80.4) | 52 (86.7) | 16 (84.2) |

ALL, acute lymphocytic leukemia; T-ALL, T-cell acute lymphoblastic leukemia; B-ALL, B-cell acute lymphoblastic leukemia; LR, low-risk; IR, intermediate-risk; HR, high-risk.
