# Supplementary material for: KIF11 serves as a cell cycle mediator in childhood acute lymphoblastic leukemia
Source: J Cancer Res Clin Oncol. 2023 Sep 1;149(17):15609–22. doi: 10.1007/s00432-023-05240-w (PMC10620298; doi:10.1007/s00432-023-05240-w)
Supplement: Supplementary file 2 — Supplementary file2 (DOCX 33 KB) [file 432_2023_5240_MOESM2_ESM.docx]

**Supplementary Table 3. Biological pathway of GO analysis.**

| Pathway ID | GO Name/Pathway Description | Enrichment Score | *P* value | FDR |
| --- | --- | --- | --- | --- |
| GO:0051301 | Cell division | 12.59150805 | 4.54E+01 | 4.21E+01 |
| GO:0006260 | DNA replication | 8.78477306 | 4.38E+01 | 4.08E+01 |
| GO:0007067 | Mitotic nuclear division | 8.638360176 | 2.99E+01 | 2.71E+01 |
| GO:0000082 | G1/S transition of mitotic cell cycle | 5.710102489 | 2.79E+01 | 2.52E+01 |
| GO:0007062 | Sister chromatid cohesion | 5.270863836 | 2.41E+01 | 2.15E+01 |
| GO:0000722 | Telomere maintenance via recombination | 2.928257687 | 1.91E+01 | 1.66E+01 |
| GO:0006281 | DNA repair | 6.588579795 | 1.87E+01 | 1.63E+01 |
| GO:0000731 | DNA synthesis involved in DNA repair | 2.781844802 | 1.66E+01 | 1.42E+01 |
| GO:0006270 | DNA replication initiation | 2.635431918 | 1.60E+01 | 1.36E+01 |
| GO:0000086 | G2/M transition of mitotic cell cycle | 4.39238653 | 1.39E+01 | 1.16E+01 |
| GO:0000732 | Strand displacement | 2.196193265 | 1.34E+01 | 1.11E+01 |
| GO:0006271 | DNA strand elongation involved in DNA replication | 1.756954612 | 1.28E+01 | 1.05E+01 |
| GO:0000724 | Double-strand break repair via homologous recombination | 3.074670571 | 1.19E+01 | 9.71E+00 |
| GO:0006974 | Cellular response to DNA damage stimulus | 4.685212299 | 1.06E+01 | 8.42E+00 |
| GO:0031145 | Anaphase-promoting complex-dependent catabolic process | 2.928257687 | 1.04E+01 | 8.23E+00 |

GO, gene ontology; FDR, false discovery rate.
