# Supplementary material for: KIF11 serves as a cell cycle mediator in childhood acute lymphoblastic leukemia
Source: J Cancer Res Clin Oncol. 2023 Sep 1;149(17):15609–22. doi: 10.1007/s00432-023-05240-w (PMC10620298; doi:10.1007/s00432-023-05240-w)
Supplement: Supplementary file 3 — Supplementary file3 (DOCX 32 KB) [file 432_2023_5240_MOESM3_ESM.docx]

**Supplementary Table 3. KEGG pathway.**

| ID | Description | *P* value | FDR |
| --- | --- | --- | --- |
| hsa03030 | DNA replication | 4.50E-27 | 9.22E-25 |
| hsa04110 | Cell cycle | 1.53E-23 | 1.57E-21 |
| hsa03460 | Fanconi anemia pathway | 1.83E-11 | 1.02E-09 |
| hsa03430 | Mismatch repair | 1.99E-11 | 1.02E-09 |
| hsa03440 | Homologous recombination | 6.09E-10 | 2.50E-08 |
| hsa00240 | Pyrimidine metabolism | 2.49E-08 | 8.52E-07 |
| hsa03420 | Nucleotide excision repair | 3.48E-08 | 1.02E-06 |
| hsa03410 | Base excision repair | 5.39E-07 | 1.38E-05 |
| hsa04114 | Oocyte meiosis | 6.12E-07 | 1.39E-05 |
| hsa04115 | p53 signaling pathway | 1.69E-05 | 3.47E-04 |
| hsa03040 | Spliceosome | 3.37E-05 | 6.29E-04 |
| hsa03050 | Proteasome | 6.60E-05 | 0.001128323 |
| hsa00230 | Purine metabolism | 3.66E-04 | 0.005776909 |
| hsa05169 | Epstein-Barr virus infection | 5.20E-04 | 0.007608212 |
| hsa04914 | Progesterone-mediated oocyte maturation | 9.12E-04 | 0.012459512 |

KEGG, Kyoto Encyclopedia of Genes and Genomes; FDR, false discovery rate.
