# Supplementary material for: KIF11 serves as a cell cycle mediator in childhood acute lymphoblastic leukemia
Source: J Cancer Res Clin Oncol. 2023 Sep 1;149(17):15609–22. doi: 10.1007/s00432-023-05240-w (PMC10620298; doi:10.1007/s00432-023-05240-w)
Supplement: Supplementary file 4 — Supplementary file4 (DOCX 20 KB) [file 432_2023_5240_MOESM4_ESM.docx]

**Supplementary Table 4.** **Baseline characteristics and *KIF11* gene signatures in childhood ALL patients (n=19).**

| Baseline characteristics | Case number  (n) | Median expression level of *KIF11*  (min, max) | *P* value |
| --- | --- | --- | --- |
| Gender |  |  | 0.368 |
| Male | 13 | 4.72 (1.83, 11.55) |  |
| Female | 6 | 5.63 (1.97, 7.46) |  |
| Age (year) |  |  | 0.875 |
| <1 or ≥10 | 3 | 4.72 (3.20, 5.54) |  |
| ≥1 and <10 | 16 | 5.22 (1.83, 11.55) |  |
| Fusion genes |  |  | 0.694 |
| Positive | 12 | 4.01 (1.97, 7.46) |  |
| Negative | 7 | 5.37 (1.83, 11.55) |  |
| Immunophenotype |  |  | 0.712 |
| T-ALL | 3 | 4.72 (3.20, 11.55) |  |
| B-ALL | 16 | 5.22 (1.83, 7.46) |  |
| Risk stratification |  |  | 0.549 |
| LR | 9 | 5.39 (1.83, 7.46) |  |
| IR/HR | 10 | 4.50 (1.97, 11.55) |  |
| White blood cell (×10^9^/L) |  |  | 0.893 |
| ≥50 | 5 | 4.72 (1.97, 11.55) |  |
| <50 | 14 | 5.22 (1.83, 7.46) |  |

ALL, acute lymphocytic leukemia; T-ALL, T-cell acute lymphoblastic leukemia; B-ALL, B-cell acute lymphoblastic leukemia; LR, low-risk; IR, intermediate-risk; HR, high-risk.
